# Inhibition of pathogenic tau signaling via blocking of the phosphatase-activating domain by novel small molecules

**DOI:** 10.3389/fphar.2026.1829420

**Published:** 2026-06-10

**Authors:** Raghad Nowar, Ganga Reddy Velma, Jiqiang Fu, Anam Kidwai, Gwendelyn Bauc, Martha Ackerman-Berrier, Gregory R. J. Thatcher, Scott T. Brady, Manel Ben Aissa

**Affiliations:** 1 Department of Pharmaceutical Sciences, UIC Retzky College of Pharmacy, University of Illinois Chicago, Chicago, IL, United States; 2 Department of Anatomy and Cell Biology, University of Illinois at Chicago, Chicago, IL, United States; 3 Department of Pharmacology & Toxicology, R Ken Coit College of Pharmacy, University of Arizona, Tucson, AZ, United States

**Keywords:** glycogen synthase kinase 3β, neuroprotection, phosphatase-activating domain, small-molecule inhibitor, tau, tauopathies

## Abstract

**Introduction:**

Tau pathology is a major feature of Alzheimer’s disease (AD) and multiple other adult-onset neurodegenerative diseases. Aberrant exposure of an N-terminal phosphatase-activating domain (PAD) is characteristic of pathological tau, representing a toxic gain of function. Exposure of the PAD in pathological tau leads to dysregulation of protein phosphatase 1/glycogen synthase kinase 3 (PP1/ GSK3β) signaling, inhibition of fast axonal transport, synaptic dysfunction, and altered transcription, along with other pathological consequences. Previous studies showed that TNT1, an antibody against the PAD, blocked toxicity of pathogenic forms of tau.

**Methods:**

In this article, we describe a high-throughput screen for small molecules that block TNT1 binding to the PAD in an AlphaLISA screen and bind specifically to the PAD in surface plasmon resonance assays. Candidate PAD ligands (PADis) were identified, and initial biochemical and biophysical optimization produced PADis with increased affinity and selectivity. Three candidate PADis were evaluated in neuronal (rat E18 embryonic cortical neurons) and non-neuronal cells (HEK293T human embryonic kidney cells) using a nano-bioluminescence resonance energy transfer (nanoBRET) assay to assess PP1 binding and cell toxicity.

**Results and Discussion:**

All three compounds prevented PP1 binding to PAD and neurite degeneration due to pathological tau in primary cultured cortical neurons. The final candidates had an IC_50_ value between 10 and 20 nM in neurons with low cytotoxicity, CC_50_ > 75 μM in primary cultured neurons, and 40–100 μM in non-neuronal cells. PADi treatment of primary cultured neurons transfected with pathogenic tau restored axonal growth and prevented neurodegeneration. These studies establish a novel approach to therapeutics for Alzheimer’s disease and tauopathies.

## Introduction

Tau pathology is a major component of neurodegenerative diseases such as Alzheimer’s disease (AD), frontotemporal dementias (FTDs), and other tauopathies, including Pick’s disease, progressive supranuclear palsy, corticobasal degeneration, and frontotemporal dementia with Parkinson’s disease (FTDP-17) (Kneynsberg et al., 2017). Tau pathology is also a component of various other neurodegenerative conditions (Kimura et al., 2018; Simic et al., 2016), including chronic traumatic encephalopathy (Mufson et al., 2016), and by definition, tau pathology is a component of AD. Tau pathology is traditionally defined as the aggregation of tau, which is thought to be driven by tau hyperphosphorylation, leading to the release of tau from microtubules and the subsequent loss of microtubules. This formulation of the role of pathogenic tau in neurodegeneration has dominated tau research for many years; however, no effective tau-based therapies have been developed based on this understanding of tau pathology.

Recent studies have identified a different pathogenic mechanism for tau pathology. The N-terminus of tau, containing the 17-amino-acid (aa) domain (MAEPRQEFEVMEDHAGT), was discovered to be necessary and sufficient to produce a toxic gain of function for hyperphosphorylated, misfolded, and mutant pathological forms of tau (Combs et al., 2021; Kanaan et al., 2011; Lapointe et al., 2009; Morris et al., 2021). This discovery identified a novel therapeutic target for addressing pathological tau. The N-terminal sequence comprises a phosphatase-activating domain (PAD) that is normally sequestered in tau under physiological conditions; however, under pathophysiological conditions, this sequence is exposed. This was demonstrated by reaction with a specific antibody (TNT1) raised against the PAD of tau and observed in AD and multiple other tauopathies (Combs and Kanaan, 2017; Kanaan et al., 2011). Aberrant exposure of the PAD activates protein phosphatase (PP1), which, in turn, activates glycogen synthase kinase 3 (GSK3β). Active GSK3β can phosphorylate tau, along with the light chain of kinesin and other cellular targets, leading to increased tau aggregation and inhibition of fast axonal transport, which results in a dying back axonopathy, as well as presynaptic dysfunction (Brady and Morfini, 2017; Moreno et al., 2016; Mueller et al., 2021).

Significantly, aberrant exposure of the PAD has been shown to represent a consistent marker of pathological tau that is increased for every tauopathy examined (Combs and Kanaan, 2017; Combs et al., 2016; Mufson et al., 2016). The ability of pathological tau to activate axonal signaling pathways that can inhibit axonal transport and affect other neuronal functions such as synaptic transmission led us to propose that aberrant exposure of the PAD and consequent activation of the PP1/GSK3β signaling pathway is a primary toxic gain of function in tauopathies.

Recent studies established that the PAD directly interacts with PP1 to activate the enzyme and TNT1 blocks PAD interaction with PP1 (Combs et al., 2021), which prevents activation of the PP1/GSK3β signaling pathway. This suggested that targeting the PAD domain would be an effective therapeutic strategy. Antibody-based approaches to treat tau pathology with antibodies directed against various epitopes have failed in early clinical trials due to the lack of efficacy and side effects (Mullard, 2021). This includes an antibody targeting the N-terminal of tau (Monteiro et al., 2023). These studies have largely been predicated on the ability of antibodies to bind extracellular tau seeds and prevent propagation. Although efforts to develop strategies using antibodies to tau continue (Gaikwad et al., 2024), antibodies have limited access to intracellular proteins in the brain and have shown limited success in treating neurological conditions, particularly given that treatment is likely to be required over decades. This study focuses on a fundamentally different mechanism for tau pathology: the exposure of a biologically active domain in pathological forms of intracellular tau.

There are substantial advantages to small-molecule therapeutics in treating neurological diseases. Oral administration and brain bioavailability are readily achieved with small-molecule therapeutics, and such strategies are better tolerated by patients. The ability to optimize small molecules for oral and brain bioavailability with predictable pharmacokinetics (PK) is well established. Therefore, identification of a small molecule that binds PAD and mimics TNT1 in blocking PAD–tau downstream signaling would have potential therapeutic benefit. We hypothesized that the PAD:TNT1 protein–protein interaction (P-PI) could be used to develop a robust bioassay suitable for high-throughput screening (HTS) in order to identify a small molecule that blocks downstream signaling initiated by PAD–tau.

## Methods and materials

### Reagents and antibodies

The TNT1 antibody (catalog #MABN471) was purchased from MilliporeSigma (Billerica, MA). The 5A6 antibody (Antibody Registry ID: AB_528487) was obtained from the Developmental Studies Hybridoma Bank (DSHB). AlphaLISA streptavidin donor beads (#6760002S), anti-human IgG acceptor beads (#AL103 M), nickel chelate donor beads (#AS101D), and AlphaLISA pre-formulated assay buffer 10X (AL000C), as well as ProxiPlate-384 plus white microplates (#6008280), were all supplied by PerkinElmer Inc. (Waltham, MA). The competitive inhibitor peptide AEPRQEFEVMEDHAGT and its biotinylated counterpart (biotin–Ahx–AEPRQEFEVMEDHAGT) used for the PAD–peptide assay were synthesized by the UIC Research Resource Center protein core.

### Constructs

DNA constructs were generated in a pCMV backbone. The high-throughput screens used a plasmid with insert of an N-terminal tau fragment, 6D, a tau isoform formed *in vivo* through alternative splicing of exon 6, which lacks the microtubule-binding repeats and the C-terminal region of tau and therefore cannot form aggregates (Andreadis, 2005; Kanaan et al., 2012; Lapointe et al., 2009). 6D tau cannot form aggregates but constitutively exposes the PAD (Kanaan et al., 2011; Lapointe et al., 2009). For high-throughput screening, 6D tau with a C-terminal His_6_-tag for purification was produced in *Escherichia coli* using the pT7c plasmid as described previously (Kanaan et al., 2011). Cells were harvested 72 h post-induction, lysed by sonication, and clarified by centrifugation. His_6_-tagged proteins were purified using immobilized metal affinity chromatography (Talon resin, cat. No. 635502, Clontech), buffer-exchanged into assay buffer (25 mM HEPES pH 7.4, 150 mM NaCl, 0.01% Tween-20), and stored at −80 °C until use.

For the nano-bioluminescence resonance energy transfer (nanoBRET) studies, the HaloTag (Promega) was attached to the N-terminal end of the PP1γ. The NanoLuciferase tag (Promega) was attached to the C-terminal end of the WT and P301L tau constructs. P301L tau constitutively exposes the PAD in both monomeric and aggregate forms (Combs et al., 2021). These constructs were generated in the Kanaan laboratory as part of a collaborative effort. Additional single-point mutations were generated using the manufacturer’s instructions for the Quikchange Lightning Site-directed Mutagenesis Kit (Agilent Technologies), as mentioned in the protocol above. Additional plasmids used were provided by the Kanaan group and were expanded using the DNA prep kit ZymoPURE II Plasmid Maxiprep Kit (D4202). Some plasmids were generated using single-point mutagenesis, while others were generated by GenScript USA.

#### High-throughput screening assay development

**FIGURE 1:**
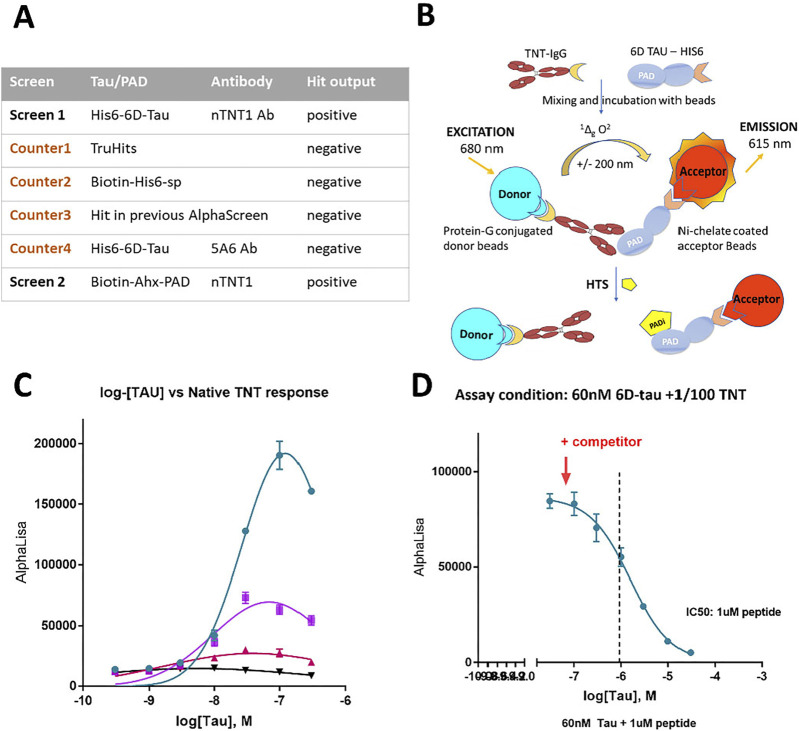
High Throughput Screen for PADi. **(A)** Tabulation of screens and counterscreens used to identify candidate PAD ligands (PADLs). **(B)** Schematic of primary assay selected and optimized for HTS. The yellow shape represents small molecules that are PADi, disrupting the P-PI between the PAD of 6D-Tau and TNT1, to inhibit the P-PI between 6D-Tau and TNT1 (an antibody that binds tau at the PAD epitope). **(C)** Titration of assay components to identify optimal assay conditions for the primary screen. **(D)** Titration against PAD peptide inhibition of the P-PI between 6D-tau and TNT1 antibody.

We assembled a 17,000-compound PPI-focused library comprising a 10,000-member subset from ChemDiv and 7,000 compounds from TargetMol. The approach used to identify inhibitors of the PPI between the exposed pathogenic tau–PAD and PP1 used, as a surrogate, inhibition of the PPI between tau–PAD and the PAD-binding TNT antibody. Therefore, we developed an assay measuring the PPI between His_6_-tagged 6D (with its PAD domain exposed) and the TNT antibody, miniaturized for 384-well plates and evaluated, optimized, and validated using the signal-to-background ratio and Z′-factor (Figure 1). Detection was performed using the AlphaLISA™ system (PerkinElmer). His_6_-tagged 6D Tau (50 nM) was incubated with the native TNT antibody (25 nM) in 20 µL assay buffer. After 1 h at room temperature, 5 μg/mL nickel-chelate donor beads and 5 μg/mL Protein G acceptor beads were added and incubated for 30 min in the dark. The AlphaLISA signal (615 nm) was read on a BioTek Synergy multimode microplate reader. Compounds were screened at 10 μM, and hits were defined as those causing ≥50% signal reduction relative to DMSO controls. A non-tagged PAD–Tau peptide (100 nM) served as a competitive in-plate inhibitor control.

#### Counter-screening to eliminate assay interference

To minimize false positives, we implemented three orthogonal counterscreens: (1) *TruHits interference assay*: PerkinElmer’s TruHits kit (catalog #AL904C) was used, according to the manufacturer’s instructions, to identify compounds that quench singlet oxygen or a fluorescence signal. (2) *Bead interference assay*: A biotin–His_6_ 16-amino-acid peptide was incubated with the native TNT antibody in combination with streptavidin donor beads and nickel-chelate acceptor beads to detect compounds that disrupt nickel-chelate bead interactions. (3) Pan-assay interference compounds (PAINS) *and cross-screen filter*: Hit compounds were cross-referenced against an internal PPI-screen database using the same bead combination to flag any PAINS.

#### Orthogonal and selectivity assays

Selected hits were further triaged in two additional tau-based PPI AlphaLISA™ assays: (1) 5A6 antibody assay: His_6_-tagged 6D tau (50 nM) was paired with the 5A6 anti-tau antibody (25 nM). The AlphaLISA signal was generated using nickel donor and anti-IgG acceptor beads. Compounds that inhibited the PPI with both the TNT and 5A6 antibodies were deprioritized as displaying non-specific inhibition of PAD. (2) PAD-peptide assay: A 16-amino-acid PAD peptide (100 nM) biotinylated via a six-carbon linker was incubated with the TNT antibody (25 nM), streptavidin donor beads, and protein G acceptor beads. PAD inhibitors selectively reduced the signal in this PAD-peptide PPI assay. All assays were run in triplicate. Z′-factors consistently exceeded 0.65 in all screened plates, and signal-to-background ratios were >10. Data were analyzed in Excel and Prism 9.

#### Surface plasmon resonance binding studies

All experiments were performed on a Biacore 3000 (GE Healthcare) at 25 °C using HBS-EP + running buffer (10 mM HEPES pH 7.4, 150 mM NaCl, 3 mM EDTA, and 0.005% P20). (1) PAD-peptide immobilization: a streptavidin-functionalized CM chip (Xantec SAHC-1000M) was primed with running buffer; then, biotinylated PAD-peptide (150 nM in PBS +0.005% IGEPAL) was injected over channel 2 for 6 min at 10 μL/min, yielding ∼5,400 RU. (2) 6D immobilization: The 6D protein construct was immobilized on channel 1 by standard amine coupling (EDC/NHS activation, 6D at 50 μg/mL in 10 mM sodium acetate pH 4.5) to achieve ∼4,500 RU; a blank reference surface was prepared on the adjacent flow cell. (3) Analyte injection: Hit compounds were diluted in running buffer to concentrations ranging from 50 nM to 10 µM. Compound solutions were injected in separate experiments over either the 6D-coupled or PAD-peptide surfaces at 50 μL/min for 2 min, followed by a 5-min dissociation phase. (4) Surface regeneration: Between each analyte injection, the surfaces were regenerated with a 30-s pulse of 10 mM glycine (pH 2.0) at 50 μL/min, followed by re-equilibration in running buffer. (5) Data analysis: Sensorgrams were double-referenced (subtracting both blank flow cell and buffer-only injections) and fitted to a 1:1 Langmuir binding model using BIAevaluation software to derive kinetic rate constants (k_on_ and k_off_) and the equilibrium constant K_D_.

### Human embryonic kidney (HEK) cell culture and transfection

HEK293T cells (ATCC cat. No. CRL-3216) were grown at 37 °C and 5% CO_2_ in cell culture media: DMEM + L-glutamine (Gibco, #11-995-065), 5% FBS (Atlanta, #S11150), and 1% penicillin–streptomycin (Gibco, #15070063). In a 100-mm cell culture dish (Corning® tissue-culture treated culture dishes, # CLS430165), HEK293T cells were plated and maintained in the incubator at 37 °C and 5% CO_2_. Cells were at 80%–90% confluency during transfection; DMEM + L-glutamine (Gibco), 5% FBS, and 1% penicillin/streptomycin culture media was replaced by 7 mL optiMEM media (Gibco, #31985). Tau-NanoLuc and PP1γ-Halo DNA stock were prepared at the optimum predetermined concentration ratio (1:100, see the *Donor saturation assay*) in 1.5 mL optiMEM media and mixed gently. Lipofectamine 2000 (Lipofectamine™ 2000, #11668030) in 1.5 mL optiMEM was prepared, mixed gently, and incubated for 5 min. Lipofectamine mix (1.5 mL) was added to the prepared DNA stocks (1.5 mL) and incubated for 15 min. The Lipofectamine-DNA mixture (total vol 3 mL) was added to cells in preexisting 7 mL optiMEM dropwise with even distribution (using a 5-mL serological pipette). Cells were incubated at 37 °C in a CO_2_ incubator, and after 4–6 h, media was replaced with new 10 mL media and incubated for 18 h. Cells were then detached using 0.5% trypsin and re-plated onto poly-L-lysine (PLL; Sigma #P1399)-coated 96-well plates (Greiner Bio-One CELLSTAR 96-well, Cell Culture-Treated, Flat-Bottom Microplate, #07-000-138) at a density of 40 × 10^3^ cells/well. HEK293T cells used in these assays were obtained from ATCC, and the maximum number of passages was 10.

### Animal use and approvals

All studies using rats were conducted at the University of Illinois at Chicago according to guidelines established by NIH and AAALAC for use of rodents in research. The protocols are reviewed annually by the Institutional Animal Care and Use Committee of UIC. The most recent review and approval is covered by the ACC Protocol Number 24-158, which includes all procedures utilized in the current study.

### Primary neuronal culture and transfection

Our standard methods of culturing primary cortical neurons were used (Morris and Brady, 2022). Pregnant Sprague Dawley rats were obtained from Charles River Laboratories, and embryos were collected on embryonic day 18 (E18). Rats were anesthetized via intraperitoneal injection with ketamine (100 mg/kg)/xylazine (5 mg/kg) and decapitated while under anesthesia. Embryos were placed on ice to anesthetize them and decapitated; then, cortices were dissected from both hemispheres and placed in ice-cold 1X Hanks Balanced Salt Solution (HBSS; Gibco #14185052). Cortical hemispheres were dissociated in trypsin (Gibco #15140148) (15 min at 37 °C) and then washed three times in 1x HBSS. Dulbecco’s modified Eagle medium (DMEM; Gibco #11995065) supplemented with 10% fetal bovine serum (FBS; Gibco #16000044) and 1X penicillin/streptomycin (Pen/Strep; Gibco #15140122) (DMEM++) was added to deactivate the trypsin; then, the tissues were triturated into a single-cell suspension with a flame-polished glass Pasteur pipette. Cell numbers were determined using a hemocytometer, and the cell solution was diluted to 1–2 × 10^4^ cells/well in DMEM++. For analysis, neurons were plated onto 6-well or 8-well μ-Slide VI 0.4 (Ibidi, #80606) chamber slides that had been coated with 0.5 mg/mL poly-L-lysine (PLL; Sigma #P1399) in 0.1 M borate buffer and 10 μg/mL laminin (Invitrogen #23017015) and then incubated in a sterile humidified incubator at 37 °C with 5% CO_2_. After 4 h, 50% of the media was replaced with Neurobasal™ Medium (Gibco #21103049) supplemented with 1X B27™ Plus (Gibco #A3582801), 1X Pen/Strep, and 0.5 mM GlutaMAX™ (Gibco #35050061) (NB+++). The neurons were maintained at 37 °C with 5% CO_2_, with 50% of the media replaced with fresh NB+++ every 3–4 days throughout the experiment.

To transfect primary cortical neurons, we used NeuroMag transfection reagent (OZBiosciences, #NM50500) following the manufacturer’s instructions. In brief, transfection was conducted at day 7, with a plasmid for the appropriate tau construct. The constructs included either a C-terminal mCherry tag or NanoLuc tag. Transfection reagents combined 1 μL NeuroMag reagent with 0.4 μg DNA in 20 μL unsupplemented neurobasal medium, followed by incubation for 15 min at room temperature. At that point, culture media was removed from a well, mixed with transfection reagent, and then added back onto the wells. Cell culture plates were placed onto a magnetic plate (OZBiosciences, #MF10000) for 15 min at 37 °C to facilitate uptake. Post-transfection, cells were maintained for a specified interval before fixation for immunocytochemistry.

The magnetofection protocol was also used to transfect primary neurons for nanoBRET assays. In these assays, neurons were plated and transfected in 96-well plates (Greiner Bio-One CELLSTAR 96-well, Cell Culture-Treated, Flat-Bottom Microplate, #07-000-138), coated as described above with poly-L-lysine (PLL; Sigma #P1399) laminin (Invitrogen #23017015) prior to plating.

Plated cells were incubated in a sterile humidified incubator at 37 °C with 5% CO_2_ for 4 h, after which 50% of the media was replaced with Neurobasal™ Medium (Gibco #21103049) supplemented with 1X B27™ Plus (Gibco #A3582801), 1X Pen/Strep, and 0.5 mM GlutaMAX™ (Gibco #35050061) (NB+++). Cells were maintained in a sterile humidified incubator at 37 °C with 5% CO_2_ throughout the experiment, with 50% of the media replaced with fresh NB+++ every 3–4 days. On Day 7 of primary culture, neurons were transfected using the NeuroMag protocol as described above with plasmids for Tau-NanoLuc and PP1γ-Halo (1:40) in a final volume of 100 μL/well.

### Nano-bioluminescence resonance energy transfer assay

The binding of ligands to the PAD and the ability to prevent interactions between the PAD domain in tau and PP1γ were evaluated using the nanoBRET assay system (Combs et al., 2021). This assay measures changes in the fluorescence signal produced when the donor tau-NanoLuc interacts with the Halo-PP1 acceptor. The NanoLuc signal produces light at 450 nm, which excites the halo tag (bound to HaloTag® NanoBRET® 618 Ligand, Promega #G9801) when it is within 10 nm, leading to emission of light from the Halo at 618 nm. The signal was optimized using a donor saturation assay, in which a constant level of donor DNA was co-transfected with different levels of acceptor DNA to determine when the fluorescence signal was maximized. Optimal conditions were established for both HEK293T cells and primary cultured cortical neurons from the rat brain. Cells were transfected as described above, then trypsinized, collected, and resuspended in 618 ligand-treated media (1:1,000 dilution). Cells were then plated and allowed to attach overnight at 37 °C and 5% CO_2_. Both luminescence (450 nm) and fluorescence (618 nm) signals were measured. Six replicate wells with 40,000 cells in 100 µL media per well (HEK 293T cells) or 15,000 cells/well (primary cortical neurons) were used for each condition. The cells were incubated for 18–20 h at 37 °C and 5% CO_2_. A 5× stock solution of Nano-Glo substrate (Promega, # RN1571) was prepared by performing a 1:100 dilution of the provided substrate into OptiMEM (no phenol red, 4% FBS); an aliquot of 25 μL of the Nano-Glo substrate stock was then added to each well and immediately placed in the plate reader (SpectraMax i3x Microplate Reader). After shaking the plate for 30 s, the luminescence values were read at 450 nm (donor signal) and 610 nm (acceptor signal). The mean corrected nanoBRET ratios were calculated. The raw milliBRET ratio was calculated by dividing the acceptor fluorescence values by the donor luminescence values and then multiplying by 1,000. These values were then corrected by subtracting the control milliBRET ratios (with DMSO) from the experimental milliBRET ratios (with fluroescent ligand). The mean and SD of these values were then plotted versus the transfection DNA ratios and fitted to a hyperbolic curve using GraphPad Prism.

Once transfection was optimized, ligands of interest were added to the assay at different concentrations through serial dilutions to generate dose–response curves and calculate IC50. HEK293T cells or primary cortical neurons were plated as described above onto white, clear-bottom 96-well plates. In brief, nine serial dilutions of test compounds were prepared to a final concentration of 100 µM down to 0.015 µM in media containing 0.1% DMSO. The media in each well was replaced with 100 µL of treatment media with compounds at a specified dilution, and a tenth well contained control media with 0.1% DMSO but no compound. Twenty-four hours later, Nano-Glo substrate was added to each well as described above; then, luminescence (donor) and fluorescence (acceptor) signals were measured. The mean corrected nanoBRET ratios were calculated as described above using a SpectraMax i3x microplate reader. The raw milliBRET ratio was calculated by dividing the acceptor fluorescence values by the donor luminescence values and then multiplying by 1,000. IC50 values were calculated using a four-parameter nonlinear regression model (variable slope, constrained top and bottom) in GraphPad Prism. Additional IC_50_ mean values were obtained from all three independent experiments (n = 3) ± SD.

### Cell toxicity assays for test compounds

Cytotoxicity was evaluated using the CellTox™ green cytotoxicity assay (#G8471, Promega). This assay measures changes in membrane integrity due to cell death in both HEK293T and primary neuronal cells treated with test compounds. HEK293T or neuronal cells were cultured in a 96-well black clear-bottom tissue culture plate (Invitrogen™ #M33089) at a density of 30,000 and 15,000 cells/well, respectively, and incubated overnight to allow the cells to adhere to the surface before treating with test compounds for 24 h. All wells were treated with test compounds in a threefold dilution series starting from 100 μM to 0.005081 μM final concentration overnight. CellTox™ green reagent was prepared according to the manufacturer’s instructions, and 50 μL was added to all experimental wells. The plate was mixed briefly using an orbital shaker (500–700 rpm) and kept at room temperature for 10 min. Fluorescence was measured at 485 nm (excitation) and 520 nm (emission) using a SpectraMax i3x microplate reader. CC50 values were calculated using a four-parameter nonlinear regression model (variable slope, constrained top and bottom) in GraphPad Prism to calculate CC_50_ mean values obtained from three independent experiments (n = 3) ± SD.

### Axonal degeneration assay

Axonal degeneration assays were conducted as described previously (Morris and Brady, 2022). In brief, primary cortical neurons were cultured as described above in 8-well μ-Slide VI 0.4 (Ibidi, #80606) chamber slides at 5,000–10,000 cells/well. Chambers were pre-coated with poly-L-lysine (PLL; Sigma #P1399) and laminin (Invitrogen #23017015). After plating, cells were maintained as described in the previous section, and on day 7, each well was transfected with 0.4 µg 6DTau-mCherry or the mCherry control plasmid overnight. Twenty-four hours after transfection, test compounds or vehicles were added to treatment wells at 10 µM and incubated for another 24 h in the presence of PADi ligands or vehicles. Neurons were fixed using 4% PFA, followed by immunostaining with primary antibodies, anti-tubulin (β3-tubulin or DM1a), and secondary antibody with AlexaFluor 488. Following immunocytochemistry, neurons in Ibidi chambers were imaged using confocal microscopy on a Zeiss LSM 710. Images were taken using a 40× or 63× objective with oil immersion. In studies for live cell imaging, cells were treated as described previously (Brady et al., 2024), with the live cell neuronal marker NeuO (NeuroFluor™ NeuO; STEMCELL TECH. #01801) at 24 h instead of being fixed.

Analysis of axonal degeneration was conducted by measuring the total length of neurites and the length of the longest neurite of treated and untreated neurons using the ImageScience NeuronJ plugin for ImageJ. All measurements were conducted by individuals blinded to the experimental condition. All living transfected cells (judged by nuclear stain, tubulin integrity, and mCherry content) that were contained completely within an image were numbered in ImageJ (n = 15 per well, with n being the number of neurons measured in four wells per treatment group). Merged images were then split into individual channels, and the tubulin channel was evaluated using the NeuronJ plugin. The measurements were collated, and an average of 15 neurites was measured, across four wells, for each treatment group, per experiment in three different rats. The following variables were analyzed for each neuron: total length of all neurites on a neuron and length of the longest primary neurite.

### Statistical analysis

The only data subjected to statistical analysis were the axonal degeneration assays. Sample size was determined empirically based on prior studies in our laboratory on axonal degeneration [see, for example, Morris and Brady (2022)]. Measurements were made by individuals blinded to the experimental condition of the images, and all data collected were used. The data were consistent for each condition, and no outliers were noted or excluded. No formal test for outliers was performed. Data normality was assessed using Q-Q plots and the Kolmogorov–Smirnov test. Normally distributed data were analyzed using a parametric t-test.

Variables (total neurite length or length of longest neurite) in the axonal degeneration assays were analyzed using an unpaired t-test, with significance set at p < 0.05. Each compound was evaluated as an individual experiment, and no statistical comparisons were made between compounds. Axonal degeneration data were analyzed using both a parametric t-test (*p* < 0.0001 for FU-2-2 and FU-2-64, and p = 0.0035 for FU-1–165) and the Mann–Whitney test with resulting p-values <0.0001 for FU-2-2 and FU-2-64, and *p* = 0.0032 for FU-1-165. The results were essentially the same in both tests. For the live neuron study, comparisons among the vehicle and Tau-6D transfected with or without FU-2-64 were evaluated using one-way ANOVA with Sidak’s multiple comparisons. All statistical comparisons and graphs were generated in GraphPad Prism 9.0.0.

## Results

The PAD:TNT1 PPI was used to develop a robust bioassay suitable for HTS to identify small molecules that could block downstream signaling initiated by PAD-tau. We established screens and counterscreens to identify small molecules from a chemical library biased toward compounds with properties likely to benefit disruption of PPIs. The optimized primary screen used an AlphaLISA assay (Beaudet et al., 2008) that was validated for HTS. After hit identification and validation, direct binding of hit compounds to the PAD was confirmed using surface plasmon resonance (SPR)-based assays. As shown below, validated hits were evaluated to test whether these PAD ligands could prevent binding of protein phosphatase, PP1. Previous studies had shown that PP1 binding to the PAD activated the phosphatase and initiated a kinase signaling cascade via GSK3β activation (Combs et al., 2021; Morris and Brady, 2022). PP1–PAD binding to PP1 was evaluated using a novel nanoBRET assay in HEK293T cells and primary cultured cortical neurons, showing that these PADis block the interaction of PP1 with the tau PAD, which is required for the activation of the PP1/GSK3 signaling pathway by pathological forms of tau.

### Overall bioassay strategy to identify PADi

Considering the importance of the interaction between PAD and PP1 in activating the PP1/GSK3β signaling cascade and the emergence of P-PI as a promising new class of therapeutic targets, we developed a 384-well plate assay capable of measuring TNT1 binding to PAD-tau. We used the AlphaLISA assay technology (Beaudet et al., 2008) to detect and discover inhibitors of the PAD/TNT1 PPI in a high-throughput format and incorporated counterscreens and secondary screens in the bioassay workflow (Figure 1A).

### Assay development

To evaluate the potential application of AlphaLISA technology in detecting PAD/TNT1, we selected a 6D-Tau construct as it constitutively displays the PAD domain that is sequestered in normal tau. 6D tau is a noncanonical N-terminal isoform comprising amino acids 1–143 plus 11 unique C-terminal amino acids (Luo et al., 2004), which lacks the proline-rich domain, microtubule-binding repeats, and C-terminus. Tau 6D does not form aggregates, but it constitutively exposes the PAD as it is normally sequestered through interactions with the C-terminus (Nowar, 2023). Plasmids for tau 6D proteins were expressed in *E. coli* and purified to purity >95%. To facilitate their detection in AlphaLISA assays, 6D proteins were appended with various tags (His, GST, Halo, or Myc tag). All PPI assays were independently optimized using the respective recombinant proteins (data not shown). The AlphaLISA assay was optimized to ensure that the signal was stable and reproducible with optimal CV values of <6% for intra-assay reproducibility and <20% for reproducibility between assays.

To determine optimal protein concentrations for the primary AlphaLISA PPI assay, we performed a cross-titration of each 6D-tau protein construct in the presence of varying concentrations (from 0.1 nM to 300 nM) of its binding partner, the TNT1 antibody. The maximum signal was achieved at 100 nM His6-6D-Tau when captured with nickel chelate donor beads in combination with 60 nM native TNT1 IgG antibody captured with Protein G AlphaLISA acceptor beads (Figure 1B). To ensure that the assay conditions were below the “hook point” (Figure 1D), we identified the optimal working conditions for the primary assay as His6-6D-Tau (60 nM) and TNT1 (40 nM) and estimated a K_D_ of ≈12 nM for this PPI pair. We further tested the 6D-tau/TNT1 P-PI assay against increasing concentrations of a 16 aa PAD peptide competitor. The peptide competitor successfully inhibited this P-PI with an IC50 of ≈ 1 μM (Figure 1D).

### High-throughput screen methodology

Using the optimized AlphaLISA methodology and binding partners, it was possible to develop robust HTS-compatible assays that exceeded the minimum pass criteria (Z′ >0.5, SW > 2, and CV <20%). Thus, our primary 6D-tau/TNT1 PPI assay optimized in a 384-well format yielded a Z′ factor >0.75. Having established the conditions and suitable performance for the primary PPI assay, we screened 10,000 compounds at a final compound concentration of 10 µM (n = 1) using a ChemDiv PPI library containing compounds with H-bonding and other characteristics biased toward PPI inhibition and omitting PAINS.

Selection of primary actives was based on a threshold for compounds that yielded a decrease in the normalized assay AlphaLISA signal >50%. A total of 584 initial hits were identified for the primary AlphaLISA PPI assay (5.8% hit rate). This hit rate includes active compounds that are false positives arising from artifactual activity primarily associated with the AlphaLISA assay components.

### Counterscreen for hit refinement

**FIGURE 2:**
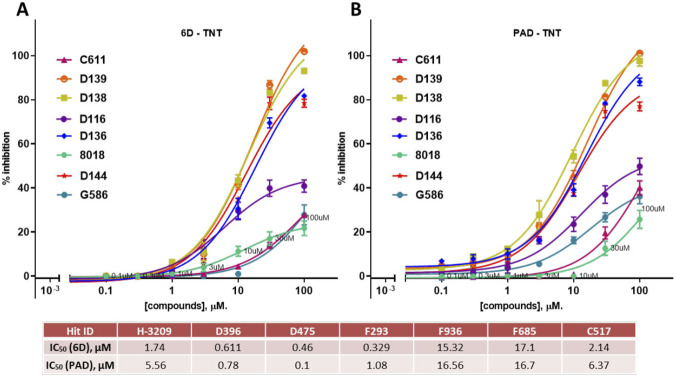
Identification of Candidate PADi. Examples of data for hit validation showing concentration-response for inhibition of the interaction between TNT1/6D-Tau **(A)** and TNT1/PAD **(B)** measured using AlphaLISA Screen assay. Data shows mean and SEM from triplicate measurements. Potency data obtained from concentration-response are tabulated for selected hits.

In the case of the AlphaLISA technology, false positives can be caused by color quenching, interference of singlet oxygen quenching, auto-fluorescence, and disruption of the interaction of the hexahistidine tag used in this PPI assay. Therefore, we first eliminated (1) compounds that caused a median decrease in the normalized AlphaLISA signal >40% of similar AlphaLISA PPI screens run in-house (data not shown; also see Figure 1A) and (2) actives that produced artifacts with the AlphaLISA technology as identified using the AlphaLISA TruHits assay. We next implemented in our workflow an additional AlphaLISA-screen assay that incorporated a biotinylated hexahistidine peptide to allow screening for interference compounds and further eliminate compounds that interfere with Ni-chelate beads prevented the binding of the 6D-His6-tag to the capture beads in the PPI complex (Figure 1A).

Of the initial actives, 93 were selected as unique to the 6D-based AlphaLISA PPI assay forming our first pool of potential hit compounds. To identify target-specific positives and investigate the selectivity and promiscuity of PPI inhibitors to PAD, we further triaged our first pool of active compounds against a 6D-based AlphaLISA PPI assay that serves as a counterscreen, measuring the interaction between the 5A6 antibody and its binding partner His6-6D (as used in the primary screen). In this PPI counterscreen, we selected the 5A6 antibody as it recognizes an N-terminal 6D-tau epitope outside the PAD domain starting from aa 19. Compounds that inhibited this interaction are not PAD-selective and were considered negatives and eliminated from the pool of validated hits for progression (Figure 1A). This counterscreen led to progression of 26 hits that were selected and retained for further investigation.

### Hit validation, secondary screen, and biophysical assay

The hit rate was approximately 0.25% prior to further validations and confirmation of binding by SPR. Hit compounds capable of interfering with 6D-tau/TNT1 and selective for PAD binding were prioritized for repurchase, retesting, and validation using a 7-point concentration–response curve (Figure 2A). We next developed a secondary PPI screening assay to confirm the selectivity for PAD and further triage our pool of validated hits as candidate PADi ligands. The AlphaLISA PPI assay was developed and optimized to measure the interaction between the TNT1 antibody used in the primary HTS assay and a 16-amino-acid peptide containing residues 2–17 of the PAD peptide in place of 6D-tau that was used in the primary screen (Figure 1A). The PAD peptide was biotinylated at the N-terminus via introduction of a 6-aminohexanoic acid (Ahx) spacer.

This secondary screen should replicate observations in the primary PPI assay to identify PAD-selective PPI inhibitors. The secondary assay was optimized using similar procedures to those used for the primary screen. Streptavidin AlphaLISA donor beads were used to capture the PAD peptide (60 nM), and protein G acceptor beads were used to capture the IgG TNT1 antibody (40 nM 1/100). A good correlation was observed between responses in primary and secondary assays for the majority of hits (Figures 2A,B). After inspection of the dose–response curves, 12 compounds were prioritized as PADi candidates. The hit rate, at this stage, was approximately 0.12%.

Having determined that hits validated in the primary assay were effective in disrupting the PPI with the PAD peptide alone, we proceeded to test for another unwanted mechanism of PPI inhibition. Disruption of the PPI in both primary and tertiary assays can occur via indirect binding to tau or via binding of hits to TNT1. To select for hits that bind to PAD-tau, SPR was used with His6-6D-tau immobilized on a nitrilotriacetic acid (NTA) and/or a CM5 chemical modification sensor chip (Supplementary Figure S1). TNT1 was used to validate the assay, giving K_D_ = 1.28 ± 0.12 µM (NTA) and K_D_ = 2.71 ± 0.10 µM (CM5) for binding to 6D-tau, which is in accord with AlphaLISA measurements (Supplementary Figure S1). Only hits that gave binding sensorgrams in the biophysical SPR assay, reflecting binding to tau, were progressed for further study.

#### Early hit-to-lead development

**FIGURE 3:**
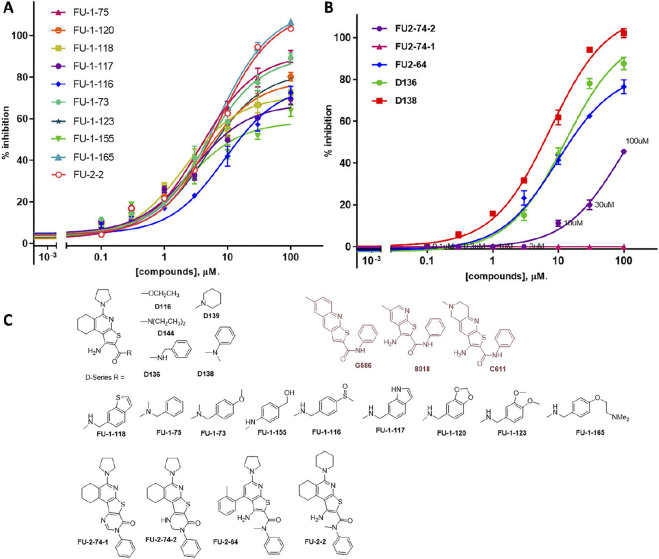
Candidate PADi compounds. **(A,B)** Concentration-response for selected Fu- and D-series hits and analogues in the primary AlphaLISA Screen P-PI assay. **(C)**. Sample structures of effective compounds.

From the validated hits, the “D-series” of 3,6-diamino-thieno[2,3-b]pyridines were selected for further study based on potency and efficacy but primarily on the observation of multiple hits within this chemical series and structurally related hits, represented by G586, 8018, and C611 (Figure 3). Of the purchased analogs and re-synthesized hits, the 2-carboxylate ester, D116, had lower efficacy. Therefore, we synthesized further analogs to explore the 2-carboxamide position (Figure 3). FU-1-116 had lower potency possibly because the polarity of the sulfoxide substituent and bicyclic substituents showed lower efficacy (Figure 3). Compounds FU-1-165 and FU-2-2 gave full inhibition of the binding of 6D-tau to TNT1, and both FU-1-73 and FU-1-75 showed similar potency with reduced efficacy (Figure 3). Conformational locking by cyclization is a standard approach in lead optimization. Cyclization of the exocyclic 3-amino group in derivatives FU-2-74-1 and FU-2-74-2 led to a complete or significant loss of activity (Figures 3B,C). Replacement of the aliphatic ring with an aromatic substituent in FU-2-64 led to a minor loss of activity, and replacement of the pyrrolidine with piperidine in FU-2-2 produced a PADi candidate with similar activity to that of FU-1-165. Although these compounds represent early leads amenable to further optimization, FU-1-165, FU-2-2, and FU-2-64 were advanced to test for additional cell-based target engagement.

#### Target engagement in cells

**FIGURE 4:**
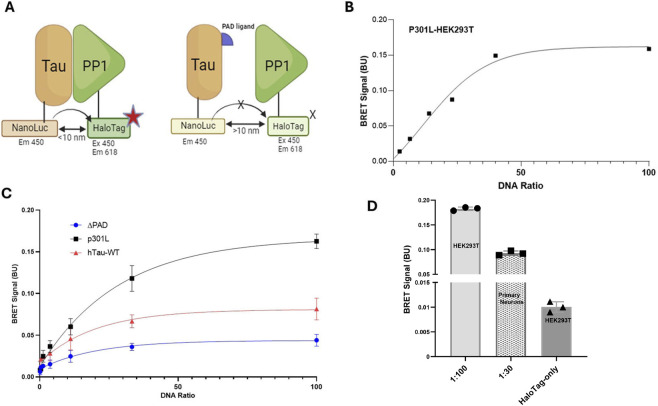
NanoBRET assay development. **(A)** Schematic illustrating the Nano Bioluminescence Resonance Energy Transfer (nanoBRET) assay components and the effect of PADi binding to the PAD-tau. **(B)** The acceptor:donor DNA ratio was varied to generate a donor saturation assay curve in HEK293T cells. The donor and acceptor signals were measured using luminescence and reported as nanoBRET ratio in BRET units (BU). Donor saturation experiments showed differential signal curves with P301L and hTau-WT. The amplitude of the signal was greatest for the forms of tau displaying the PAD domain. **(C)** Donor saturation experiments were performed using a tau construct without the PAD domain (Δ PAD) as control for the PAD engagement in generating the nanoBRET signal. **(D)** Optimum Tau/PP1γ transfection ratios were selected from donor saturation experiments in both HEK293T and Primary neurons. These ratios were found to be 1:100 and 1:30, respectively. Plots show the data from three replicates for each condition and the error bars show standard deviation.

Cell-based assays are required to test for target engagement in a biologically relevant cellular context and to confirm data obtained from cell-free/biochemical assays. Based on the PPI biochemical assays, three of the most promising compounds, FU-1-165, FU-2-2, and FU-2-64 were evaluated for the ability to block PP1γ binding to the PAD in cells. We developed a nanoBRET assay (Figure 4) to identify the PP1 isoform that is activated by PAD exposure and established PP1γ as the physiologically relevant isoform (Combs et al., 2021). We adapted the nanoBRET assay for evaluating the ability of compounds to inhibit PP1γ binding to PAD in both HEK293T and primary cultured rat neurons. In brief, cells were doubly transfected with recombinant PP1γ with a Halo tag as an acceptor and a PAD-containing polypeptide linked to a nano-luciferase as a donor (Figure 4). When the PP1γ is bound to the PAD, the nano-luciferase excites the Halo tag to fluoresce at 618 nm. This requires the two components to be within 10 nm of each other. In the presence of a PADi that disrupts this interaction, there is little or no fluorescence detectable (Figure 4A; Supplementary Figure S3).

To optimize the nanoBRET assay for both HEK293T cells and primary cultures of cortical neurons, we determined the levels and ratios for transfection by plasmids for the PP1γ Halo acceptor and different tau donor constructs. The PAD in wild-type hTau40 (hTau-WT) is generally sequestered and exhibits a low baseline level of nanoBRET activation, consistent with the structural dynamics of tau. In contrast, a mutant tau associated with frontotemporal dementia (P301L) that has an exposed PAD (Combs et al., 2021) exhibits elevated activity in both HEK293 cells (Figure 4B) and primary cultured cortical neurons (Figure 4C). The P301L tau constitutively exposes the PAD and is toxic in both monomer and aggregate forms (Combs et al., 2021). This activity is dependent on the presence of a PAD domain, where a tau construct from which the PAD is deleted (delta PAD) exhibits minimal activation (Figure 4C). Optimal transfection levels for HEK293T cells and primary neurons were determined (Figure 4D). Two pathological forms of tau were used in assays of tau ligand binding, 6D tau and P301L tau. Both of these have been shown to produce constitutive exposure of the PAD and to activate PP1 and GSK3 in cells and axoplasm (Combs et al., 2021; Kanaan et al., 2011; Nowar, 2023). The initial 12 hits were initially tested in the nanoBRET assay to demonstrate target engagement of PADi with the tau-PAD and validate target engagement of PADi and the tau PAD in these cells to prevent binding between PP1γ and the PAD (Nowar, 2023).

#### Cell-based assessment of target engagement and efficacy

**FIGURE 5:**
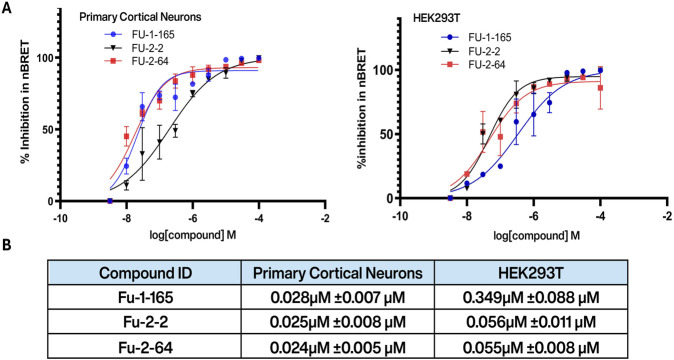
NanoBRET dose-response curves and IC_50_ values for candidate PADi ligands. **(A)**. NanoBRET Dose–response curves in primary neurons (left) and HEK293T cells (right) transfected with P301L tau. Values represent percent inhibition of the NanoBRET signal at each compound concentration. Data points represent mean ± SD of two technical replicates (n = 2) from a single independent experiment, selected as representative of three independent experiments. **(B)**. IC_50_ values were calculated using a four-parameter nonlinear regression model (variable slope, constrained top and bottom) in GraphPad Prism. Additional IC_50_ mean values obtained from all three independent experiments (n = 3) ± SD for HEK293T are Fu-1-165= [0.349μM ±0.088]; Fu-2-2 = [0.056 μM ± 0.011]; Fu-2-64 = [0.055 μM ± 0.008]; for Primary neurons: Fu-1-165 = [0.028 μM ±0.007]; Fu-2-2 = [0.025 μM ± 0.008]; Fu-2-64 = [0.024 μM ± 0.005].

Using the optimized nanoBRET assay with mutant tau (P301L) as the donor and PP1γ as the acceptor, we evaluated the ability of the three ligands identified as the best candidates in biochemical assays to inhibit the PP1γ/tau interactions in HEK293T cells and primary cultured rat cortical neurons (Nowar, 2023). All studies utilized paired assays that compared results with and without ligand. The results were expressed as the ratio of the signal with a ligand to the signal without a ligand at different concentrations from 10^−8^ to 10^−4^ M to generate dose–response curves for the ligands. All three final candidate compounds significantly inhibited the interaction between tau and PP1γ with potency 10–100 nM in primary cortical neurons and HEK293T cells (Figure 5A). Despite the presence of competing endogenous rat tau in primary neurons, potency was comparable in both cell cultures. In primary cortical neurons, FU-2-2 and FU-2-64 were the most potent PADi tested.

#### Cytotoxicity of candidate compounds

**FIGURE 6:**
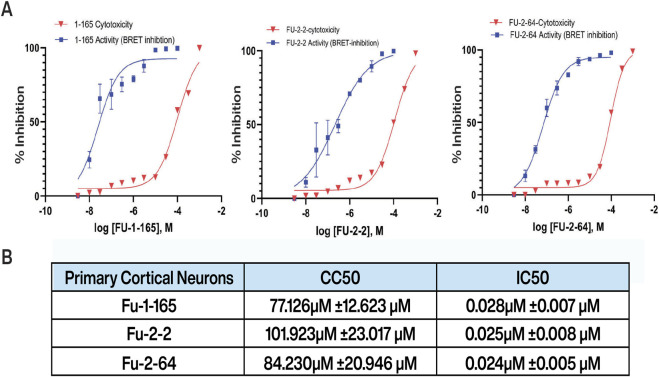
Cytotoxicity of PADi in Primary Cultured Neurons. **(A)** Comparison of concentration-dependence for each of the three PADi ligands in efficacy with the nanoBRET assay (red curve) and cytotoxicity using CellTox™ Green assay (blue curve). **(B)** Comparison of CC_50_ and IC_50_ values for each of the three candidate PADi ligands.

Since the nanoBRET assay is a loss-of-signal assay, cytotoxic compounds could conceivably lead to artifacts. The toxicity of the three PADi ligands was tested in both HEK293T cells (Supplementary Figure S2) and primary cultured rat cortical neurons (Figure 6) using the CellTox™ green cytotoxicity assay. All three compounds were well tolerated at concentrations that blocked the interaction between tau and PP1γ. The CC50 was >3 orders of magnitude greater than measured IC50. This observation supports the interpretation of the nanoBRET assay as demonstrating cell-based target engagement. The lack of cytotoxicity in primary cell cultures also supports further testing in primary neurons for efficacy and potentially future development for therapeutic use in treatment of tauopathies. This raised the question of whether these compounds are neuroprotective in cellular models of tauopathy.

#### Neuroprotection by PADi ligands

**FIGURE 7:**
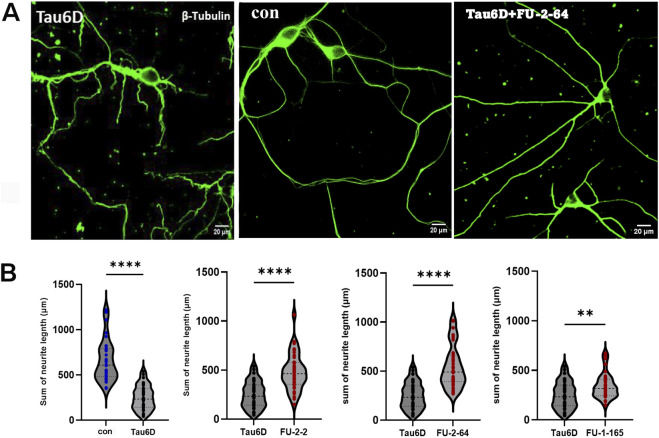
Rescue of degenerating neurites in neurons expressing pathogenic tau by PADi. Primary cortical neurons were cultured on poly-L-lysine + laminin for 7 days before transfection with pathogenic Tau-6D or Vehicle (Veh) and incubated overnight before addition of PADi. **(A)** Representative images of primary cultured neurons. Neurons were fixed and processed for immunofluorescence at 48 h after transfection. Neurons were stained with the neuronal marker β3-tubulin (green). After 24h, neurons transfected with Tau-6D exhibit degenerating neurites while neurites are intact in neurons transfected with mCherry only vectors (con). Treating neurons with Fu-2-64 at 24 h after transfection recues degenerating neurites. **(B)** Plots shows sum of total neurite length comparing effects of 6D tau transfection to vehicle, while experiments with each of the candidate PADis exhibit significantly increased neurite length, indicating full or partial reversal of Tau-6D induced damage. Data represents 12 replicates across 3 different rats for each condition (n = 36). Neurons transfected with pathogenic tau (6D) exhibited neurodegenerative changes and a significant reduction in total neurite length (*p* < 0.0001, t = 9.014, df = 66) when compared to vehicle-only control neurons (con) in a two tailed unpaired t test. Each of three PADi compounds were effective in significant increasing total neurite length compared to the 6D transfected vehicle group using a two tailed unpaired t test. FU-2-2 (*p* = <0.0001, t = 5.947, df = 70) and FU-2-64 (*p* < 0.0001, t = 6.895, df = 70) were the most effective PADi at rescuing neurites. FU-1-165 (*p* = 0.0035. t = 3.021, df = 70) was somewhat less effective but still significantly improved over 6D Tau alone. The sum of lengths of ∼5 neurites per neuron were measured across 12 different images for each treatment group in multiple wells from cultures from 3 different rats. Violin plots show the distribution of data, where the width reflects the underlying density of the data at each level. The center line represents the median of the data, while the lower line indicates the level of the first quartile and the upper line indicates the third quartile. The extreme ends of the plots correspond to the largest and smallest data point in the sample. Data normality was assessed using Q-Q plots and the Kolmogorov-Smirnov test. The results were found to be normally distributed (see Supplemental Figure S4). All statistical comparisons and graphs were generated in GraphPad Prism 9.0.0.

Having demonstrated target engagement and explored analog-by-catalog SAR, we initiated preliminary hit-to-lead optimization of one series of PADi. This limited optimization was intended to demonstrate feasibility and proof of concept, so we tested several candidate PADis in cell cultures. To determine whether the three candidate compounds can prevent neurodegeneration in a cellular model of tauopathy, we transfected primary cultured neurons with a pathogenic fragment of tau, 6D-tau (Kanaan et al., 2011; Lapointe et al., 2009) (Supplementary Figure S5). The 6D form of tau is a noncanonical isoform of tau comprising the N-terminal 143 amino acids, with an additional unique C-terminal of 11 amino acids resulting from alternative splicing at exon 6 (Luo et al., 2004). Tau 6D lacks the proline-rich domain, microtubule-binding repeats, and the C-terminus, so tau 6D cannot form aggregates. The PAD is constitutively displayed in tau 6D, and this construct is toxic in axonal transport assays (Kanaan et al., 2011; Lapointe et al., 2009) and when expressed in neurons (Figure 7). Data normality was assessed using Q-Q plots and the Kolmogorov–Smirnov test (Supplementary Figure S4).

Neurons were transfected at 7 DIV with 6D tau, and after 24 h, either the vehicle or the candidate compound was added at 10 µM. These studies were intended as a proof of concept, so a single high dose of 10 µM was chosen to avoid any problem associated with the overexpression of the pathogenic tau construct. A dose–response curve for neuroprotection will be constructed in future studies. After 24 h of transfection, neurites have begun to degenerate (Supplementary Figure S5). After a further 24 h of incubation, cells were fixed and processed for immunostaining with anti-β3-tubulin (to label neurites) and DAPI (to label nuclei) (Figures 7A,B). Adding the PADi after neurite degeneration has begun tests the ability of PADi to rescue neurons undergoing degeneration, thereby providing a more realistic model for potential therapeutic use. Vehicle-treated neurons expressing 6D for 2 days exhibit substantial degeneration of axons as the neurons begin to lose viability. In contrast, neurons expressing 6D tau treated with PAD ligands showed less axonal degeneration (Figure 7C). Each PADi compound was compared to untreated tau-6D neurons independently. Data were analyzed using a parametric t-test (*p* < 0.0001 for FU-2-2 and FU-2-64, and *p* = 0.0035 for FU-1-165.) and the Mann–Whitney test with resulting *p*-values <0.0001 for FU-2-2 and FU-2-64, and *p* = 0.0032 for FU-1-165 (Supplementary Figure S4). All three compounds showed significant neurite rescue and neuroprotection even at these short time intervals (Figure 7C), with FU-2-64 exhibiting the strongest protective effect, consistent with observations on blocking PP1γ interactions with the PAD in the nanoBRET assay (Figure 5). This result is consistent with a mechanism in which PAD/PP1 interaction is required for the pathogenic effects of the 6D Tau.

## Discussion

Tau pathology is one of the most common features of adult-onset neurodegenerative diseases, with the appearance of tau aggregation and aberrant phosphorylation present as either a primary or a secondary pathological hallmark of diseases such as Alzheimer’s disease and related neurodegenerative diseases. The association of pathological tau with disease progression stimulated interest in tau as a therapeutic target, leading to strategies for reduction in tau aggregates or inhibition of kinases implicated in hyperphosphorylation. Despite intensive efforts, these approaches have failed to generate effective therapies for treating tauopathies.

The discovery that the PAD, a biologically active motif in the N-terminal of tau, is aberrantly exposed in the pathogenic forms of tau, providing a molecular basis for the toxicity of pathological tau (Combs et al., 2019; Kanaan et al., 2011; Lapointe et al., 2009). This finding suggested a novel approach to treating tau pathology and defined a novel therapeutic target. TNT1, a specific antibody against the PAD, was neuroprotective in model systems and blocked the activation of a downstream signaling pathway involving PP1 phosphatase and GSK3β kinase (Combs et al., 2021; Kanaan et al., 2011). We used TNT1 and a pathogenic form of tau to develop a high-throughput AlphaLISA screen for identifying small molecules that would bind to the PAD and block binding of the TNT1 antibody to PAD. This strategy identified a candidate set of ligands that satisfied the criteria of displacing TNT1 antibody binding to the PAD. Biophysical characterization by SPR demonstrated selectivity to establish that these ligands acted by binding to the PAD and demonstrated suitably high affinity for the PAD.

Phenotypic cell-based assays were used for target validation and efficacy (nanoBRET) and to evaluate cytotoxicity (CellTox™ Green). These assays demonstrated that the candidate compounds exhibited target engagement and low toxicity. The final three candidate compounds exhibited IC_50_ values in the range of 20–30 nM for primary cortical neurons with CC_50_ values >75 µM, suggesting a strong therapeutic index. The most promising candidate FU-2-64 had an IC_50_ value of ∼20 nM for blocking binding of PP1 and exhibited an ability to block the effects of pathological tau compared to that of inhibitors of PP1 or GSK3β (Combs et al., 2021; Kanaan et al., 2011; Morris and Brady, 2022; Nowar, 2023). Although the possibility of additional cellular targets for the PADi was not evaluated, the ability of these compounds to prevent the binding of PP1γ to the PAD in both neuronal and non-neuronal cells indicate that any such targets did not interfere with the efficacy of PADi in blocking PP1γ interactions with tau through the PAD.

All three candidate PADi ligands were neuroprotective for neurons challenged with pathogenic tau. FU-2-64 was extensively evaluated for the ability to prevent tau-based neurodegeneration in primary cultures of rat cortical neurons (Supplementary Figure S6). When neurons undergoing axonal degeneration due to pathogenic tau were treated with a PADi, axonal degeneration was blocked, and axonal outgrowth resumed.

These studies identify a novel therapeutic target for treatment of tauopathies and describe a series of compounds that effectively block pathogenic mechanisms associated with tau pathology both *in vitro* and cultured neurons. They exhibit low toxicity and are effective in living cells. No evidence of off-target effects was observed in either neuronal or non-neuronal cells. Given the absence of effective therapies for treating adult-onset neurodegeneration with tau pathology, such as Alzheimer’s disease and related disorders, these studies define a novel approach to treat these diseases. Future studies will evaluate these compounds in preclinical models of tauopathies.

## Data Availability

Raw data generated in this study is available upon request as specified by NIH guidelines and archived https://doi.org/10.25417/uic.32337666 and additional information will be made available consistent with a patent application. Data management is in accord with current policy at NIH.
