# Supplementary material for: Inhibition of pathogenic tau signaling via blocking of the phosphatase-activating domain by novel small molecules
Source: Front Pharmacol. 2026 Jun 10;17:1829420. doi: 10.3389/fphar.2026.1829420 (PMC13291570; doi:10.3389/fphar.2026.1829420)
Supplement: Supplementary file 1 [file DataSheet1.pdf]

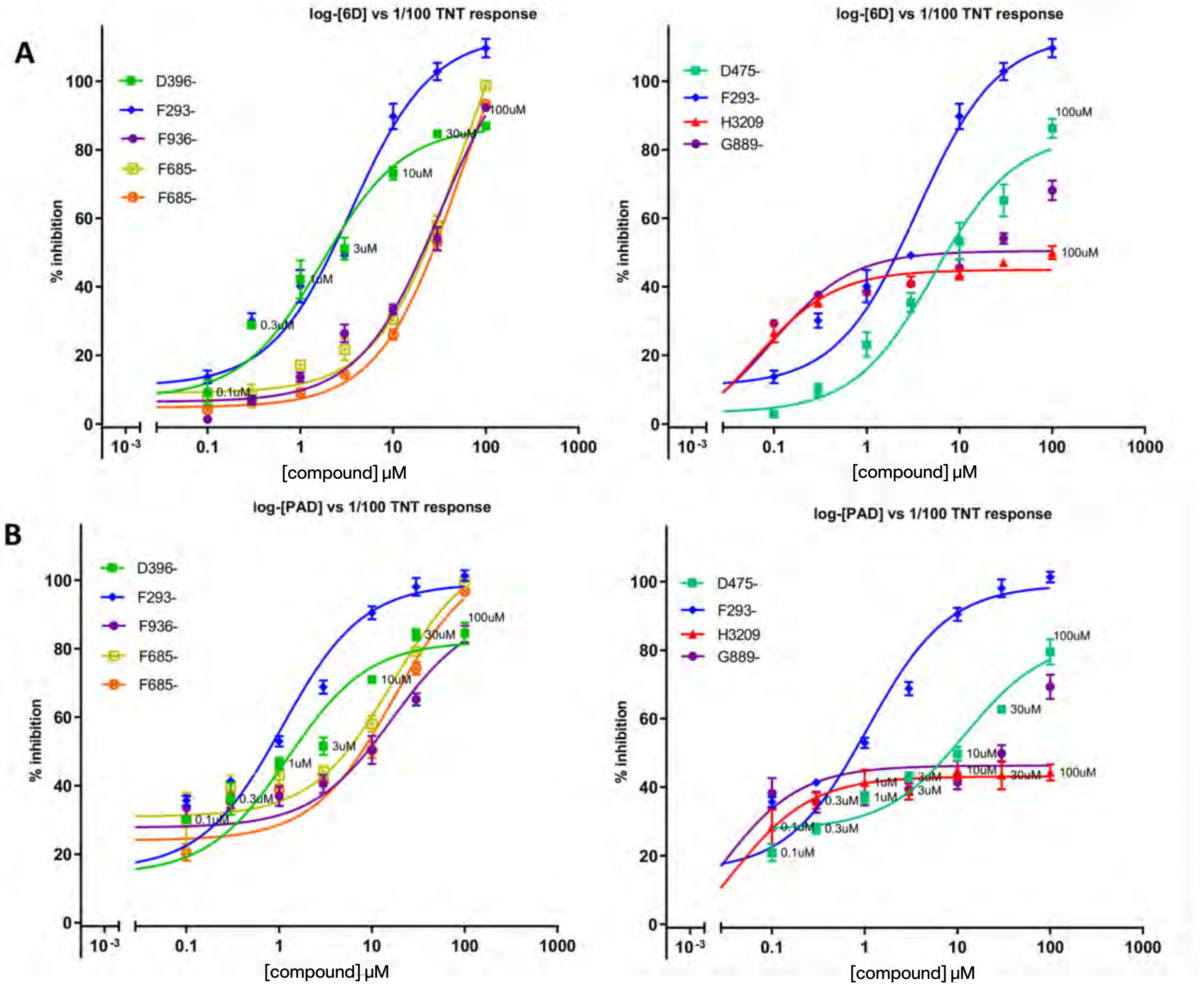

Supplemental Figure 1. Exemplar data showing concentration-response for inhibition of TNT1/6D-Tau (A) and TNT1/PAD (B) measured using AlphaScreen assays. Comparison of hits in the two assays shows strong qualitative correlation between the two assays. Data shows mean and SEM from triplicate measurements.

A

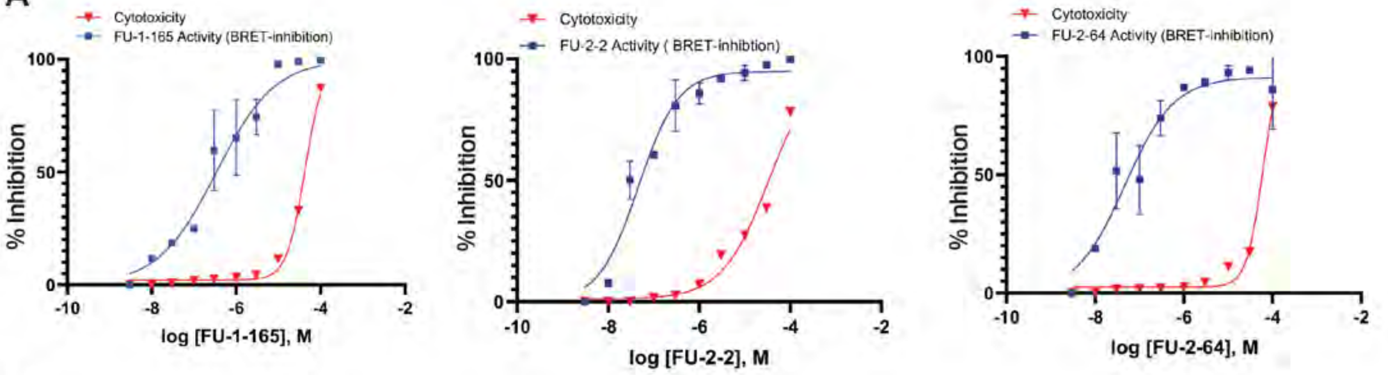

B

HEK293T	CC50	IC50
Fu-1-165	43.39 μM	0.349μM ±0.088 μM
Fu-2-2	94.70 μM	0.056μM ±0.011 μM
Fu-2-64	62.46 μM	0.055μM ±0.008 μM

Supplemental Figure 2. Cytotoxicity profiles of PADi ligands in HEK293T Cells. (A) Comparison of concentration-dependence for each of the three PADi ligands in efficacy with the nanoBRET assay (blue curve) and cytotoxicity using CellTox™ Green assay (red curve). Data points and CC_{50} values are indicated as the mean ± SD of two replicates (n = 2) using a four-parameter nonlinear regression model (variable slope) implemented in GraphPad Prism. B. Comparison of CC50 and IC50 mean values in HEK293T cells for each of the three candidate PADi ligands.

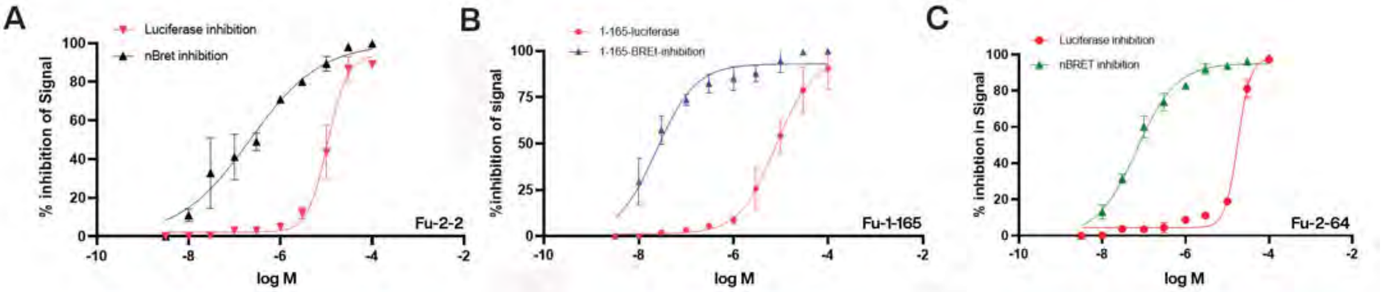

FU-2-2	
Luciferase inhibition (μM)	nBRET inhibition (μM)
~10.5	0.0462

FU-1-165	
Luciferase inhibition (μM)	nBRET inhibition (μM)
~8	0.341

FU-2-64	
Luciferase inhibition (μM)	nBRET inhibition (μM)
~17	0.0469

Supplemental Figure 3. Control for direct inhibition of luciferase activity. (A) FU-2-2, (B) FU-1-165, and (C) FU-2-64. No significant direct inhibition of luciferase was observed for the FU series. In HEK293T cells Compounds FU-2-64 and FU-1-165 showed >20-fold reduced potency in P301L-nLuc single-transfection assays (450 nm) compared with NanoBRET inhibition assays (618/450nm ratio using P301L-nLuc donor and PP1-Halo acceptor). Data and IC_{50} values represent mean ± SD (n = 2) obtained using a four-parameter nonlinear regression model in GraphPad Prism.

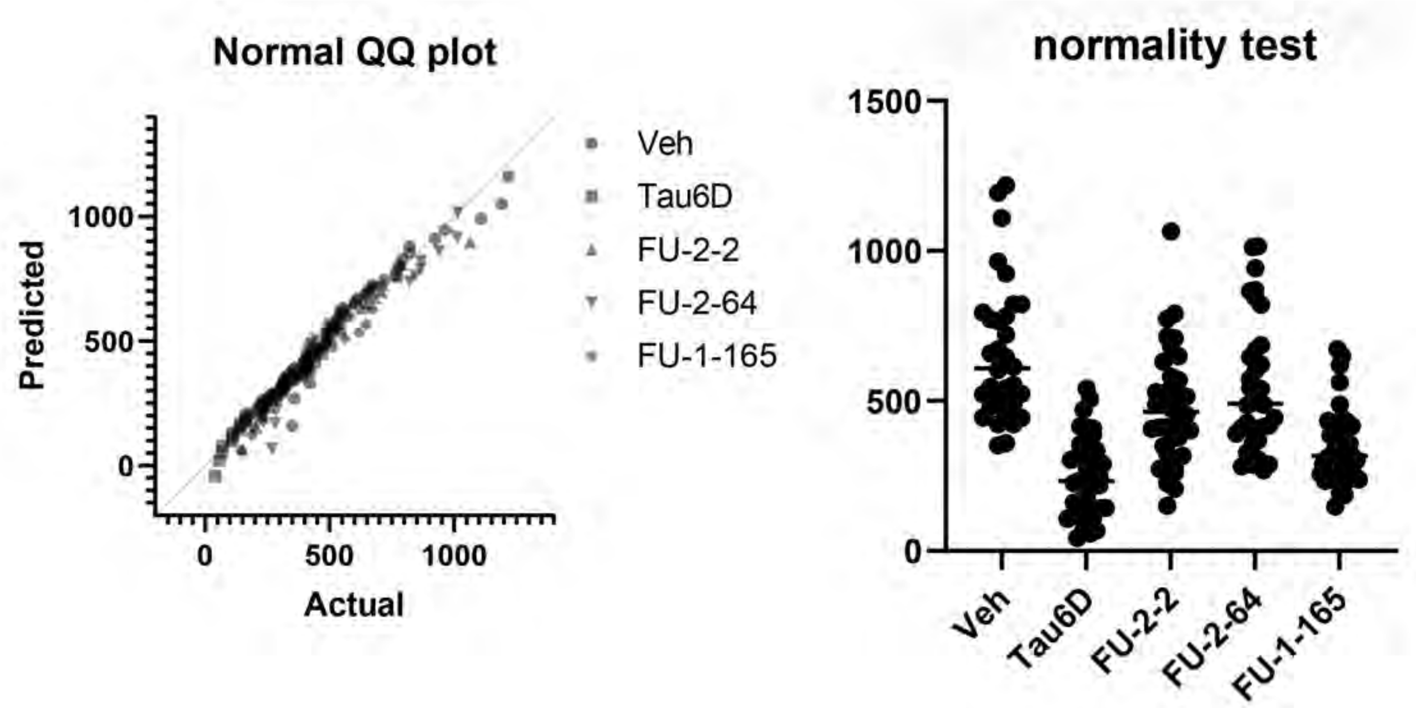

Supplemental Figure 4. The range of neurite lengths was normally distributed. The data was obtained by individuals blinded to the experimental conditions. Wild type neurons (WT) were not transfected but were treated with the vehicle used for drug treatments in parallel with treatment with PADi. For the other groups, neurons were transfected with Tau6D which constitutively exposes the PAD at 7 div, then 24 hours later were treated with the indicated PADi ligands for 24 hours before fixing and processing for immunofluorescence.

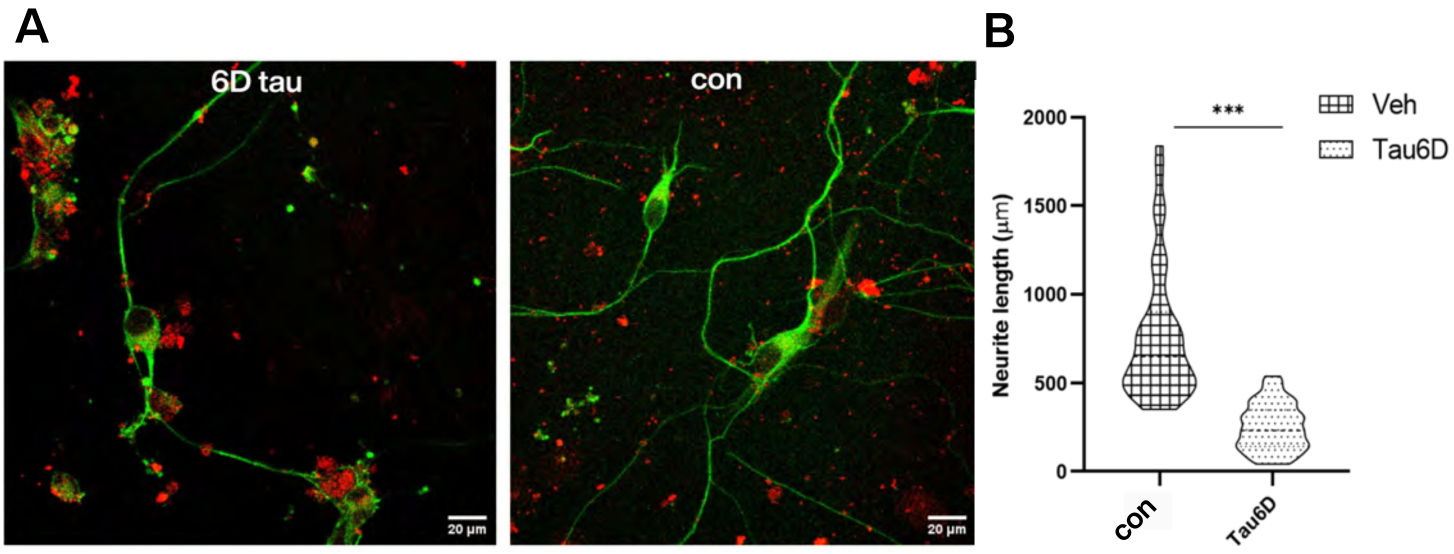

Supplemental Figure 5. 6DTau-mCherry induced axonal degeneration in primary cortical neurons. A. 6DTau-mCherry induced axonal degeneration in primary cortical neurons. Primary cultured cortical neurons are treated with 6Dtau-mCherry (6D Tau) or mCherry (con) and fixed after 24h. B. Total neurite length was measured using NeuronJ, n=36 (36 neurons per treatment group, replicated in cultures from 3 different rats). A two tailed unpaired t test indicated a significant decrease ($p<0.0001$, $t = 7.688$, $df = 70$) in total neurite length was observed with 6D treatment as compared to control group at 24 hr post transfection.

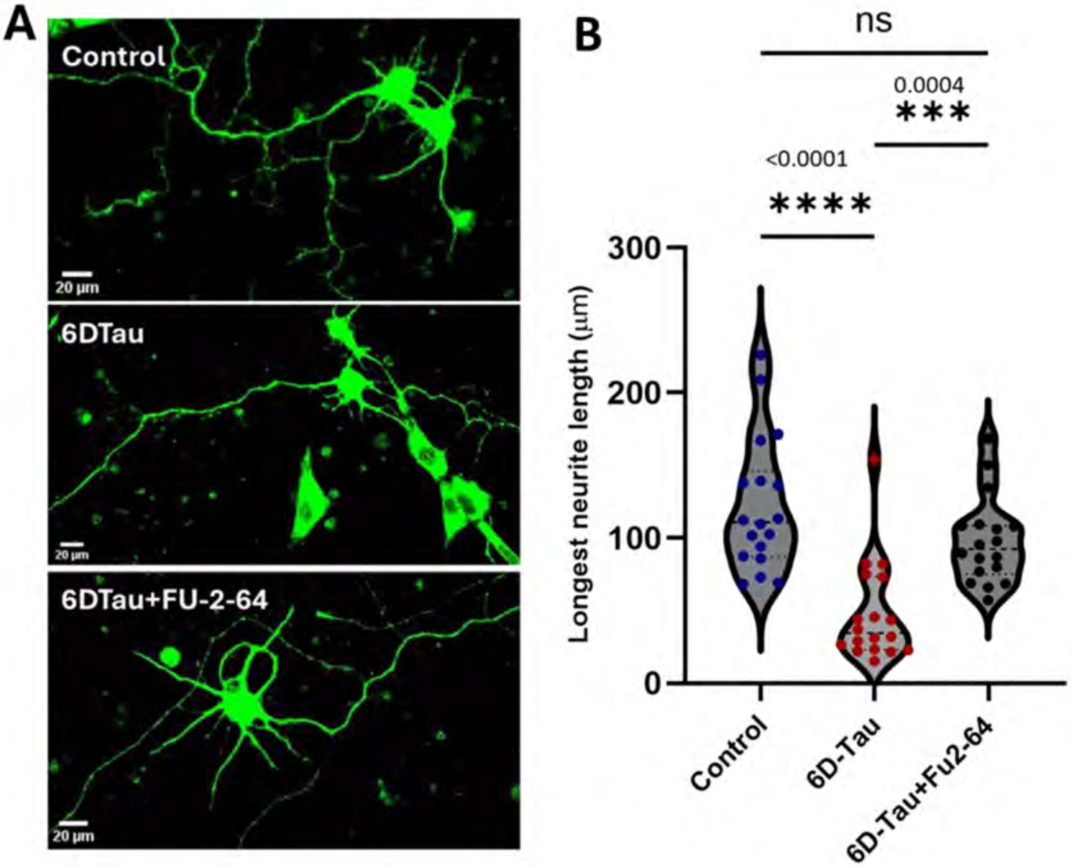

Supplemental Figure 6. FU-2-64 showed a significant increase in total neurite length and longest primary neurite length in Live Cell Imaging. A. Primary cultures of neurons were plated and maintained for 7 days before 6D-tau transfection. Twenty-four hours after transfection, neurons were treated with vehicle or with Fu-2-64 at 10μM overnight. Representative images showing the longest neurite of each of the control group, 6D-treated group and 6D+Fu-2-64 treatment. Live neurons were visualized with the neuronal marker NeuO (NeuroFluor™ NeuO; STEMCELL TECH. #01801). B. There was a significant reduction in longest neurite length for 6D tau–transfected neurons compared to control neurons ($p < 0.0001$, one-way ANOVA with Sidak's multiple comparisons, ($n = 18$), indicating significantly induced neurodegeneration. FU-2-64 treatment of 6D tau–transfected neurons significantly rescued this effect ($p = 0.0004$) and showed no significant difference from control neurons.
